# Update on PET Tracer Development for Muscarinic Acetylcholine Receptors

**DOI:** 10.3390/ph14060530

**Published:** 2021-06-02

**Authors:** Marius Ozenil, Jonas Aronow, Marlon Millard, Thierry Langer, Wolfgang Wadsak, Marcus Hacker, Verena Pichler

**Affiliations:** 1 Department of Biomedical Imaging and Image-Guided Therapy, Medical University of Vienna, 1090 Wien, Austria; marius.ozenil@meduniwien.ac.at (M.O.); jonas.aronow@meduniwien.ac.at (J.A.); wolfgang.wadsak@meduniwien.ac.at (W.W.); marcus.hacker@meduniwien.ac.at (M.H.); 2 Department of Pharmaceutical Sciences, Division of Pharmaceutical Chemistry, University of Vienna, 1090 Wien, Austria; marlon.millard@univie.ac.at (M.M.); thierry.langer@univie.ac.at (T.L.)

**Keywords:** molecular imaging, PET, tracer development, muscarinic acetylcholine receptors

## Abstract

The muscarinic cholinergic system regulates peripheral and central nervous system functions, and, thus, their potential as a therapeutic target for several neurodegenerative diseases is undoubted. A clinically applicable positron emission tomography (PET) tracer would facilitate the monitoring of disease progression, elucidate the role of muscarinic acetylcholine receptors (mAChR) in disease development and would aid to clarify the diverse natural functions of mAChR regulation throughout the nervous system, which still are largely unresolved. Still, no mAChR PET tracer has yet found broad clinical application, which demands mAChR tracers with improved imaging properties. This paper reviews strategies of mAChR PET tracer design and summarizes the binding properties and preclinical evaluation of recent mAChR tracer candidates. Furthermore, this work identifies the current major challenges in mAChR PET tracer development and provides a perspective on future developments in this area of research.

## 1. Introduction

The manifold of functions controlled by muscarinic acetylcholine receptors (mAChR) range from the involvement in peripheral and central neurotransmission [1,2] to their contribution to cancer development [3,4]. While the need for selective and specific mAChR ligands is of eminent importance and the interest for the target remains unabated, there still is a significant absence of potent and selective mAChR ligands in modern nuclear medicine.

The main area of interest for mAChR ligands for functional positron emission tomo-graphy (PET) remains the central nervous system (CNS), aggravating the radiotracer development process due to the required ability of the ligand to penetrate the blood-brain barrier (BBB) without being a substrate of efflux transporters. Since the millennium, six new potential mAChR PET tracers were evaluated in human, none of them are so far clinically established. To the best of our knowledge, the development of probes for molecular mAChR imaging has lastly been reviewed 15 years ago [5]. This motivated us to summarize and discuss its progress, with a special emphasis on recent developments.

### 1.1. Muscarinic Acetylcholine Receptors

Muscarinic acetylcholine receptors (mAChRs) are a group of G protein-coupled receptors, which bind the endogenous ligand acetylcholine and can be activated by the fungal toxin muscarine. Alongside nicotinic acetylcholine receptors, they mediate the cholinergic neurotransmission. Considering their regulatory functions in CNS, glandular secretion, smooth muscle contraction and heart rate, mAChRs adopt a pivotal role in human physiology [6]. mAChRs are divided into five subtypes (M1–M5), which are expressed throughout the central and peripheral nervous systems. Subtypes M1, M4, and M5 are mainly found in the CNS, whereas M2 and M3 also show high expression levels in the periphery [7]. Subtypes with odd numbers (M1, M3, and M5) are predominantly coupled to G_q/11_ proteins, while even-numbered subtypes preferentially signal through G_i/o_ proteins [6]. As of now the exact functional role of the different subtypes and their interplay within the same body system remains poorly defined [8]. This can partly be attributed to a lack of small molecules that can inhibit or activate specific mAChR subtypes in a highly selective fashion [6].

### 1.2. The Role of PET in Diagnosis and Therapeutic Drug Development

PET is the clinical method of choice when it comes to functional non-invasive imaging of biological processes with molecular precision [9]. For functional PET imaging, drug-target-specific radioligands are applied to (semi-)quantify pharmacological processes, such as receptor occupancy of the biological target in question [10], thereby giving insights on tissue distribution, target engagement, as well as the correlation of plasma exposure and target occupancy [11]. Although PET is mainly applied for oncological or cardiac imaging for disease staging and progression, as well as therapy monitoring in routine clinical practice [12,13], it is an ongoing process to find similar applications for neurological questions besides neuro-oncology. In this context, amyloid PET imaging using [^11^C]PIB or similar radiofluorinated tracers has taken a pioneering role [14], highlighting the importance of PET for investigational neurological pathophysiology as well as for fundamental research [15]. The mere application of nanomolar to subnanomolar amounts of a ligand, far beyond a pharmacological dosage, facilitates the use of pharmacologically active compounds translated into identical tracer molecules. Using this so-called microdosing concept greatly reduces the possibility of adverse effects or toxicity [16]. This concept is of particular relevance for the discussed mAChR ligands herein, considering their potential addictive and neurotoxic properties when applied in higher concentrations.

PET radiotracers targeting mAChRs could have an enormous impact on clinical disease management for Alzheimer’s and Parkinson’s disease and would potentially pave the way towards effective dementia therapies. Furthermore, there is also evidence that data from binding to mAChRs in the peripheral nervous system (PNS) could act as suitable biomarkers in the context of precision medicine [17,18]. The possibility to elucidate mode of action in vivo and fundamentally resolving parts of the pathophysiology of dementias would significantly facilitate human drug evaluation [19]. Still, while being essential for this endeavour and fundamental research in general, both the suitability of available mAChR PET tracers and the availability of suitable mAChR PET tracers is lagging behind.

Within the last decade, the pharmaceutical industry has discovered functional imaging as an effective and highly versatile tool for investigating the mode of action of therapeutics in humans. Especially CNS drug development programs are plagued by exceedingly high attrition rates, caused by the required highly specific drug parameters, including blood-brain barrier penetration and metabolic stability [20]. Molecular imaging techniques such as PET have been shown to be of tremendous value to the development of CNS drugs, in particular during early clinical development. A medical cyclotron and connected radiochemistry facility allows authentic labelling with carbon-11 of drug candidates, leaving the chemical structure advantageously unaltered [21]. In a preclinical setting, target occupancy data may facilitate the selection process which candidates are most promising for clinical trials [22]. Once in clinical trials, this information is used to aid clinical drug-dosing decisions. Imaging-informed decision-making can thus significantly limit the risk of adverse effects due to excess exposure and reduce the expenditure of time and money on Phase II dose-ranging studies [23,24,25]. Yet, in order to be able to fully exploit the benefits of PET imaging-based decision-making, considerable research efforts are necessary to advance the availability of radioligands functioning as golden standards. Additionally, more properly equipped PET facilities enabling authentic labelling are needed [23].

mAChRs can be imaged in vitro by various methods such as fluorescence [26], Förster resonance energy transfer [27], autoradiography [28] and immunohistochemistry [29]. These methods allow to study mAChRs on the cellular level and on isolated tissues but are less useful for in vivo imaging. Single photon emission computed tomography (SPECT) is a molecular imaging technique based on radionuclides emitting gamma radiation. Similar to PET, SPECT allows for in vivo imaging of molecular targets; however, due to its reduced sensitivity, it is less appealing for modern radiotracer development. In the early days of in vivo muscarinic imaging, SPECT radioligands like (*R*,*S*)-[^123^I]IQNB [30] and [^123^I]4-iododexetimide [31] participated in launching the research field, but to the best of our knowledge no novel SPECT tracers targeting the mAChRs have been published within the last twenty years.

### 1.3. Designing Small Molecules as PET Tracers for the CNS

Over the last decades a plethora of small molecules of PET tracers have been developed for brain imaging. Besides the well-known 2-[^18^F]FDG (2-[^18^F]fluoro-2-deoxy-d-glucose), nowadays commonly used small molecules radiotracers include, e.g., [^11^C]PIB (Pittsburgh compound B) for imaging cerebral beta-amyloid plaques [32], [^11^C]raclopride for imaging the cerebral D_2_ dopamine receptor [33], [^11^C]methionine for imaging amino acid uptake in gliomas [34] and [^11^C]DASB (3-amino-4-(2-dimethylaminomethylphenylsulfanyl)-benzonitrile) for imaging cerebral serotonin transporters [35].

A. D. Gee et al. recently summarized following design and test criteria for the development of small molecules as radiopharmaceutical vectors (Table 1) [36].

Design criteria can be evaluated prior radiolabeling and they support the identification of proper candidates for PET tracer development. Test criteria consist of properties that can be assessed by in vitro and in vivo experiments using the labeled compound to determine its potential utility as radiotracer.

For a PET tracer to be of clinical relevance, the chosen molecular target should be of clinical interest associated with relevant clinical questions. Furthermore, the target must be expressed at a sufficient concentration to allow for successful imaging. Although sensitivity is a major strength of PET imaging, review of the established radiotracers reveals that—with only some exceptions—the lowest B_max_ that can be successfully imaged is around 1 nM. Additionally, the size of the expressing tissue should at least exceed the resolution of the PET scanner (2–5 mm) to avoid underestimation of the signal through the partial volume effect [36]. The B_max_ strongly influences the required affinity of the radiotracer. With decreasing target expression levels (i.e., decreasing B_max_) the affinity must increase accordingly (i.e., decrease of K_D_) to maintain the necessary target-to-background ratio. As a rule of thumb, a minimum K_D_/B_max_ ratio of 10 should be reached to achieve sufficient contrast in vivo [37]. However, tracers with a K_D_/B_max_ ratio of 1–750 nM have previously been used for imaging [36]. The B_max_ of mAChRs in human brain was reported as 150 nM [38], but of course the target density is strongly dependent on the subtype and the brain area [39]. Ideally, the radiotracer should be fully selective, thereby binding only to the desired (sub-)target. However, if the radiotracer binds to more than one target, the location, B_max_ and the affinity to the off-target must be considered.

The radiochemistry of carbon-11 and fluorine-18 is a continuously evolving field, which has given rise to a wide variety of labeling reactions. Still, caused by the relatively short half-life, not all small molecules can be labeled with organic PET nuclides. Ease of the radiochemistry therefore remains an important factor when choosing a molecule for PET tracer development [40]. Diffusion or active transport of the PET tracer to the tissue of interest is a fundamental property in order to maximize target accessibility. Considering intravenous application, the BBB imposes a significant challenge for the target accessibility of brain radiotracers. The BBB comprises active transporters to satisfy the brain’s need for important, highly polar molecules, such as amino acids and glucose. Nevertheless, the vast majority of brain radiotracers do not fulfill the structural requirements to act as substrates for these transports and therefore rely on passive diffusion through the BBB, in this context, [^11^C]methionine and 2-[^18^F]FDG are notable exceptions. A lot of research effort has been put in predicting a drug’s likelihood of penetrating the BBB a priori. In addition to other physico-chemical properties, lipophilicity quantified by the logP value is frequently used as an indicator for BBB permeability. Several desirable logP ranges have been proposed; however, when applied to the entirety of brain permeable radiotracers there are numerous false positives and false negatives, limiting the value of this value. In fact, a recent study concludes that the topological polar surface area (tPSA), which can be calculated from any given chemical structure within seconds, displays a higher predictive power for the BBB permeability of radiotracers [41]. Additionally, efflux transporters, most notably the P-glycoprotein, can negatively affect the target accessibility of the radio-tracer [42].

### 1.4. Involvement of Computational Approaches in PET Tracer Design

While computational methods are common tools in therapeutic drug discovery and development, including techniques like protein-ligand docking studies, pharmacophore modeling or quantitative structure-activity relationships [43], their application in radiotracer development is still in its infancy. This is based on the fact that the common approach for radiotracer development, including the here discussed mAChR tracers, is screening drug libraries for high affinity compounds followed by successive authentic radiolabeling. However, a limited number of recent publications included computational methods, e.g., in silico evaluation of α-synuclein candidates [44], molecular docking studies of coumarin-triazole hybrid [45], cannabinoid receptor type 2 ligands [46] or focal adhesion kinase tumor radiotracers [47]. Munoz et al. applied 3D-quantitative structure–activity relationships for the investigation of differences in the inhibitory activity of VEGFR2 inhibitors [48]. To the best of our knowledge, in mAChR tracer development in silico receptor docking studies were applied for the development of only two novel subtype selective ligands [49,50]. The infrequent application of computational methods for radiotracer development is in stark contrast to the extensive use of computational methods in other stages of PET imaging such as highly sophisticated image post-processing. While radioligands for brain imaging and CNS-acting drugs share a range of common requirements, such as relatively high target affinity and sufficient brain penetration, they also require distinctly different properties. The most notable characteristic in this regard probably is the degree of non-specific binding. A high degree of non-specific binding, while certainly impacting pharmacokinetic, does not generally rule out a molecule’s chance of becoming a successful therapeutic drug [11]; however, it is one of the primary causes for potential PET imaging agents to fail [51,52]. Hence, the chances of repurposing a small molecule therapeutic drug as a radioligand by merely labeling it are rather small. Unfortunately, properties such as the extent of non-specific binding can only be rather modestly predicted by in vitro methods [51]. In 2008, Rosso et al. described an ab initio methodology to estimate the non-specific binding of 10 commonly applied CNS targeting radiotracers [53]. The same group applied their quantum chemical approach on further 22 compounds and found a significant correlation to the in vivo non-specific binding of the calculated compounds [52]. Therefore, we want to encourage an increasing development and use of in silico methodologies which are specifically devoted to predicting characteristics important for imaging agents to come up with novel, innovative scaffolds and subtype-selective mAChR ligands.

## 2. Development of PET Tracers for mAChRs

### 2.1. Development of mAChR Ligands

**Figure 1:**
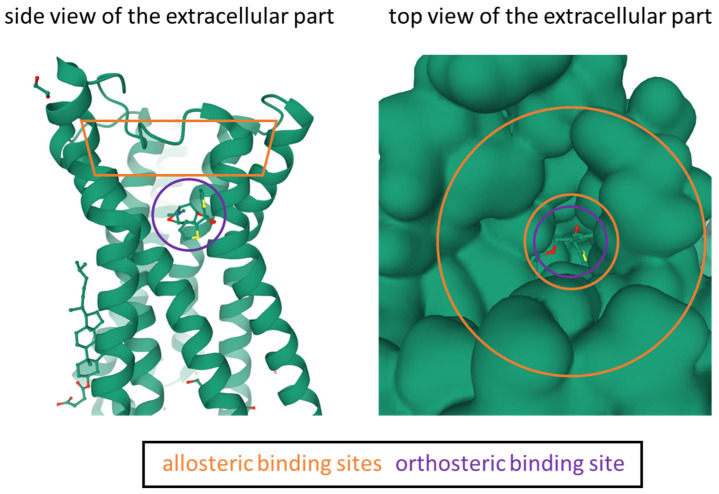
Graphical illustration of the mAChR M1 crystal structure PDB:5CXV [54] using Mol* [62]. The approximal location of the orthosteric binding site and the allosteric binding sites are highlighted. The receptor structure contains the co-crystallized orthosteric antagonist tiotropium, which is displayed in ‘ball and stick’ style. In the side view the protein is displayed as cartoon and in the top view it is displayed as Gaussian surface.

Based on their location of binding, mAChR ligands can be divided in orthosteric, allosteric, and bitopic ligands [6]. Orthosteric ligands bind to the same pocket as the endogenous ligand acetylcholine. The orthosteric binding site of the mAChRs is deeply buried within the transmembrane core as illustrated in the crystal structure of mAChR M1 (Figure 1) and is covered with a tyrosine lid [6]. In the region of the orthosteric pocket, crystal structures of the other mAChR subtypes show an overall very similar picture, which is not surprising when considering the high degree of sequence homology between the subtypes [54]. Most well-known mAChR ligands (e.g., scopolamine, atropine, 3-quinuclidinyl benzilate (QNB)) [55] bind to the orthosteric pocket. Orthosteric mAChR ligands generally bear a high affinity and a well-defined structure-activity relationship [6], which are major advantages for PET tracer development. Furthermore, tritiated orthosteric mAChR ligands are commercially available (e.g., [^3^H]NMS, [^3^H]QNB) [56], which allows for straightforward affinity determination of novel unlabeled, orthosteric ligands. Based on their pharmacology, orthosteric mAChR ligands can be divided into agonists (activation of the receptor), antagonists (competitive inhibition of agonist), and inverse agonists (induces opposite pharmacological response) [57]. Allosteric ligands bind to a different part of the receptor, compared to the endogenous ligand. The vast majority of allosteric mAChR ligands bind to the cone above the orthosteric binding pocket, when viewing the receptor from the extracellular side (Figure 1). Allosteric mAChR ligands typically feature a superior subtype selectivity, which however comes at the cost of limited receptor affinity [6]. Based on their pharmacology, allosteric mAChR ligands can be basically divided into positive allosteric modulators (PAMs, increasing the affinity of orthosteric ligands), negative allosteric modulators (NAMs, lowering the affinity of orthosteric ligands), and neutral allosteric modulators [58].

The pros and cons of orthosteric ligands versus allosteric ligands are highlighted by the comparison of the two well-studied ligands scopolamine and benzyl quinolone carboxylic acid (BQCA) (Table 2).

In an attempt to combine the favorable properties of orthosteric and allosteric mAChR ligands, the concept of bitopic ligands has recently attracted strong research interest. This class of compounds is designed to simultaneously bridge both binding sites of a single receptor and aims to achieve subtype selectivity through the allosteric binding and affinity through the orthosteric binding. Although this concept seems simple and yet ingenious, its practical implementation remains cumbersome in many cases [6]. THRX-160209 can be considered a successful example of the bitopic concept [60]. However, the chemical characteristics of this class of compounds, such as the high molecular weight, indicate poor BBB permeability. Generally, there is not always consensus in the literature whether a ligand should be considered orthosteric or bitopic. In fact, several compounds which were initially described as orthosteric ligands later were reclassified as bitopic ligands [61].

Assuming that the expression levels of the different mAChR subtypes in different areas in the human brain were known, one could estimate the required subtype selectivity to image a given brain area. However, caused by the lack of truly subtypes selective mAChR ligands, only limited information on the expression levels of the different subtypes in human brain is available. Consequently, it is hard to state which levels of in vitro subtype selectivity have to be reached to justify further development as subtype selective therapeutic or diagnostic drug. Still, this basic rule holds true: The higher the difference in K_i_ values, the better. Previous developments of mAChR radiotracers have taught us that subtype selectivity in vitro does not necessarily correlate with in vivo observations. In the light of that, it makes no sense to view in vitro mAChR subtype selectivity as an absolute criterion that must exceed a certain threshold but rather as a general predictive characteristic to evaluate candidates in the drug development process.

### 2.2. PET Tracer Development for In Vivo Muscarinic Imaging of the CNS

**Figure 2:**
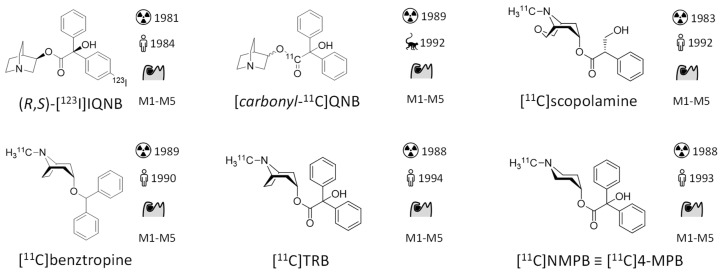

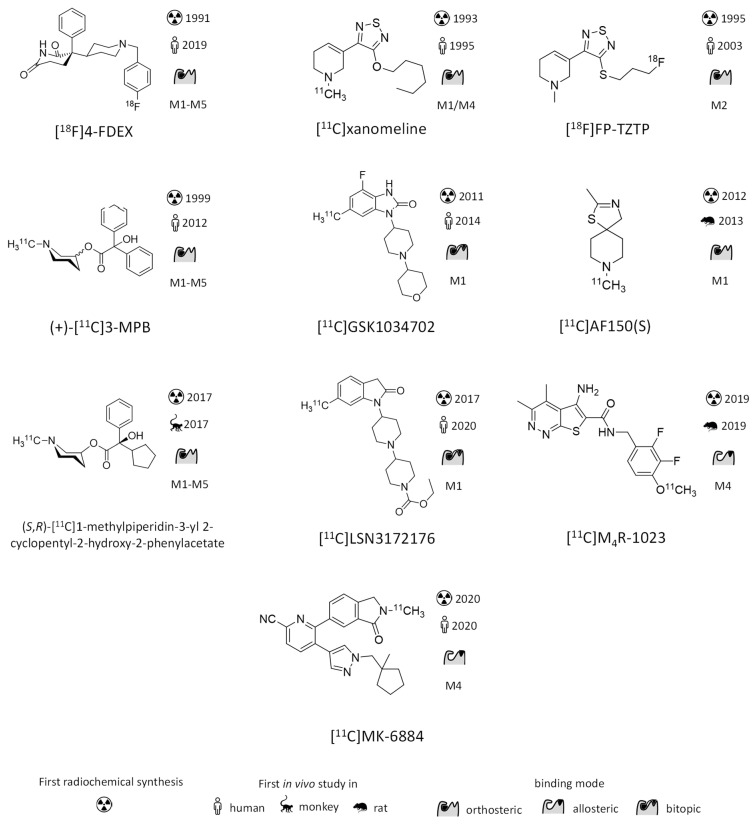
Structures and binding properties of mAChR ligands previously evaluated in vivo as imaging probes. Emphasis is given on novelties since the previous review in 2006 [5]. First synthesis: (*R*,*S*)-[^123^I]IQNB [100], [*carbonyl*-^11^C]QNB [66], [^11^C]scopolamine [101], [^11^C]benztropine [102], [^11^C]TRB [103], [^11^C]NMPB [104], [^18^F]4-FDEX [76], [^11^C]xanomeline [105], [^18^F]FP-TZTP [106], (+)-[^11^C]3-MPB [107], [^11^C]GSK1034702 [108], [^11^C]AF150(S) [92], (*S*,*R*)-[^11^C]1-methylpiperidin-3-yl)2-cyclopentyl-2-hydroxy-2-phenylacetate [94], [^11^C]LSN3172176 [109], [^11^C]M_4_R-1023 [98], [^11^C]MK-6884 [99]. First in man/animal: (*R*,*S*)-[^123^I]IQNB [30], [*carbonyl*-^11^C]QNB [110], [^11^C]scopolamine [67], [^11^C]benztropine [68], [^11^C]TRB[69], [^11^C]NMPB [70], [^18^F]4-FDEX [78], [^11^C]xanomeline [86], [^18^F]FP-TZTP [83], (+)-[^11^C]3-MPB [75], [^11^C]GSK1034702 [88], [^11^C]AF150(S) [93], (*S*,*R*)-[^11^C]1-methylpiperidin-3-yl)2-cyclopentyl-2-hydroxy-2-phenylacetate [94], [^11^C]LSN3172176 [96], [^11^C]M_4_R-1023 [98], [^11^C]MK-6884 [111].

The development of mAChR radiotracers started as early as 1982. An overview of the described mAChR radiotracers is depicted in Figure 2. (*R*,*S*)-[^123^I]IQNB was the first radio-ligand used in the mapping of central mAChRs. It is a radioiodinated version of the high-affinity chemical warfare agent QNB, which was studied extensively and stockpiled by the U.S. Army as incapacitating agent. Undoubtedly, high target affinity is a fundamental prerequisite in radiotracer development as it is the driving force to obtain the desired high target-to-background ratio in molecular imaging. However, when assuming the k_on_ to be constant, according to the kinetic definition of the equilibrium dissociation constant (K_D_ = k_off_/k_on_), high affinity comes with a slow off rate [36]. This ‘second face’ of high target affinity is assumed to compromise the in vivo radiotracer distribution of (*R*,*S*)-[^123^I]IQNB, which strongly suffered from dependence of blood flow and transport across the BBB. As a consequence, (*R*,*S*)-[^123^I]IQNB scans required two visits to the clinic and required pharmacokinetic modelling to separate the parameters of flow and transport from receptor density. Following a mAChR radiotracer like (*R*,*S*)-[^123^I]IQNB over time, the aim of mAChR in vivo imaging studies often evolves from mapping the distribution of mAChRs [30] over studying differences between healthy and diseased subjects [63] to studying the effect of diseases treatment [64]. Although promising differences in receptor density between healthy and diseased brains could be shown in the majority of clinical studies, no impact on clinical care could be delineated from these measurements. Additionally, the lack of mAChR subtype selectivity as well as the limited resolution and sensitivity of the SPECT technique hindered broad application of (*R*,*S*)-[^123^I]IQNB [5]. Nevertheless, regarding all human in vivo mAChR imaging studies from the 1980s until now, (*R*,*S*)-[^123^I]IQNB remains the most applied radiotracer [65]; however, not a single (*R*,*S*)-[^123^I]IQNB study after 2007 is reported. From a radiochemical perspective [*carbonyl*-^11^C]QNB is interesting because of its labeling procedure, which involves formation of [^11^C]benzylic acid from [^11^C]CO_2_ and the benzophenone dianion followed by esterification with 3-quinuclidinol using 1,1′-carbonyldiimidazole [66].

mAChR targeting PET tracers, [^11^C]scopolamine [67], [^11^C]benztropine [68], [^11^C]TRB [69], [^11^C]NMPB [70] developed between 1988 and 1993 suffer from similar problems: e.g. [^11^C]scopolamine showed clear differences in cerebral cortical and cerebellar uptake. However, within the measurable time frame of up to 2 h post-injection, the cerebral cortical radiotracer distribution did not correspond to the anticipated receptor densities. Analysis via kinetic modeling resulted in good agreement with expectations based on in vitro studies; however, the authors concluded that the limited precision of the method may hinder its widespread clinical application [67].

The frequently observed flow dependent radiotracer distribution shifted the goal of mAChR radiotracer development from compounds with high affinity to compounds with slightly reduced affinity, allowing to reach steady states after bolus injection or equilibrium after infusion [5]. Expression levels of the different subtypes in human brain are rarely reported; however, based on the mAChR density of M1 and M2 in human brain [71] it was estimated that a radioligand K_D_ of about 3–50 nM is suitable for imaging CNS mAChRs in vivo [72].

[^11^C]NMPB acted as lead compound for the synthesis of congeners with *N*-ethyl ([^11^C]4-EPB) and *N*-propyl ([^11^C]4-PPB) residues instead of *N*-methyl [73], which however did not advance to human studies. Similarly, regioisomers of [^11^C]NMPB regarding the piperidyl position were studied, which eventually yielded (+)-[^11^C]3-MPB as a more favorable mAChR radiotracer because of its quicker kinetic and higher specific uptake in monkey scans [74]. (+)-[^11^C]3-MPB was used to study humans suffering from chronic fatigue syndrome and showed reduced binding in the group of mAChR autoantibody positive subjects [75].

[^18^F]4-FDEX is a radiofluorinated analogue of the non-subtypeselective muscarinic antagonist dexetimide. Based on its blockable uptake in mAChR rich brain regions in mice and rats, it was suggested as mAChR tracer [76,77]. Almost 30 years later [^18^F]4-FDEX was evaluated in a first in human and dosimetry study, which showed good brain uptake (4% of injected dose at 5 min) with retention in putamen, prefrontal cortex, hippocampus and clearance from the almost mAChR devoid cerebellum [78,79]. However, to the best of the authors’ knowledge the subtype selectivity of [^18^F]4-FDEX was not evaluated.

Although studied extensively in humans, PET tracers originating in the previous millennium did rarely aim for subtype selectivity, rendering them unattractive for current clinical mAChR imaging studies. This is not caused by inadvertence in the radiotracer development, but because the existence of five different mAChR subtypes was just about to be recognized around that time [80], not to mention the unknowingness of their collective expression in human brain [81].

One of the earliest examples of a subtype selective mAChR radiotracer is [^18^F]FP-TZTP. Based on its K_i_ values for M1 (7.4 nM) and M2 (2.2 nM), [^18^F]FP-TZTP can hardly be considered subtype selective; however, comparing in vitro binding between heart and brain tissue, [^18^F]FP-TZTP exhibited M2 subtype selectivity. In vivo subtype selectivity could not be proven by the traditional pharmacological methods because of the uniform cerebral expression of M2 receptors and the lack of other M2 selective ligands which could be used for blocking experiments. Yet, ex vivo autoradiography using subtype selective knockout mice confirmed the in vivo M2 selectivity [82]. Imaging studies in monkeys applying physostigmine, an acetylcholinesterase inhibitor, revealed sensitivity of [^18^F]FP-TZTP binding to endogenous acetylcholine concentration. Consequently, [^18^F]FP-TZTP scans should be regarded as measurement of the muscarinic systems biology, rather than just M2 receptor distribution [5]. Cerebral distribution volumes of [^18^F]FP-TZTP were shown to significantly correlate with human age [83]. Its discrepancy between in vitro K_i_s and in vivo subtype selectivity is not yet fully understood but thought to be caused by a small but statistically significant slower M2 receptor off-rate [84]. This highlights the importance of studying binding kinetics in addition to binding affinity when pursuing subtype-selective mAChR radiotracer development.

[^11^C]Xanomeline and [^11^C]butylthio-TZTP were, despite their similar structure compared to [^18^F]FP-TZTP, originally described as M1 ligands. Later studies revealed an evenly good affinity of xanomeline for mAChR M4 [85]. In human imaging studies these PET ligands showed poor selectivity for mAChRs over sigma sites [86]. Similarly to [^18^F]FP-TZTP, xanomeline shows insignificant differences in in vitro K_i_ values between subtypes (Table 3) and is yet commonly considered as M1/M4 preferring ligand [87].

The mAChR M1 targeting PET radioligand [^11^C]GSK1034702 is innovative in two ways. Firstly, it is the first mAChR radioligand developed to target an allosteric binding site of a mAChR receptor. Secondly, in difference to all previous mAChR PET ligands its main goal is neither receptor mapping nor diagnosis. Instead, [^11^C]GSK1034702 was synthesized and applied in human studies for evaluating BBB permeability to de-risk the drug development process of GSK1034702 for the treatment of cognitive disorders. The observed good brain uptake of [^11^C]GSK1034702 discharged possible development risks and provided a support to advance the drug in the next stage of clinical development. Nevertheless, the authors of this study concluded that [^11^C]GSK1034702 might not be a suitable PET ligand due to the limited specific binding, which results from relatively low affinity (pEC_50_ = 8.1) or low molar activity (≈9 GBq/µmol) [88]. Although developed as an allosteric ligand, GSK1034702 was also shown to displace the orthosteric ligand [^3^H]NMS (K_i_ = 960 nM) [89] and therefore nowadays is considered as bitopic ligand [90]. A regio-isomer of GSK1034702 was recently labelled with fluorine-18, but no pre-clinical evaluation of this potential tracer was reported [91].

AF150(S) is an M1 selective mAChR agonist and was evaluated as potential therapeutic agent in animal models of neurological diseases. Despite its moderate affinity (K_D_ = 200 nM), Buiter et al. labeled the compound with carbon-11 and evaluated its use as PET tracer via rat brain autoradiography, ex vivo biodistribution and metabolite analysis in brain and blood. The authors concluded that the observed rapid metabolism, radioactive metabolites, hydrophobicity and relatively low binding affinity may be challenging for PET studies [92]. In vivo imaging studies using [^11^C]AF150(S) revealed binding enhancement in brain with increasing concentration of AF150(S) [93].

Recently, Malmquist et al. modified the structure of (+)-[^11^C]3-MPB by substituting one of the phenyl groups with cyclopentyl [94]. Based on its rapid kinetic in monkey brain, the (*S*,*R*) isomer was deemed most promising. Although the K_i_ of all four possible stereoisomers toward mAChR M1 was determined, no affinities for subtypes M2–M5 were reported. When comparing the blocking using scopolamine (not subtype selective) with the blocking using pirenzepine (M1 subtype selective) in human brain autoradiography, involvement of M2–M5 in the binding of (*S*,*R*)-[^11^C]1-methylpiperidin-3-yl 2-cyclopentyl-2-hydroxy-2-phenylacetate is evident [94].

LSN3172176 is a bitopic ligand developed by Elli Lilly, which shares several chemical features with GSK1034702. Although [^3^H]NMS displacement from M1–M5 membrane preparations attested LSN3172176 M1 selectivity, no M1 selectivity was evident when performing saturation binding using [^3^H]LSN3172176 [89]. In vivo studies in monkeys using [^11^C]LSN3172176 and pretreatment with scopolamine reduced radioligand binding to levels indistinguishable from the almost mAChR devoid cerebellum. Also, pretreatment with the M1 selective partial agonist AZD6088 strongly reduced cerebral radioligand binding but not as quantitative as scopolamine. [^11^C]LSN3172176 is rapidly metabolized in monkeys: 15 min post-injection 71% of the radioactivity is found as more polar radiometabolites. Based on its high specific brain signal paired with appropriate kinetics in rhesus monkey brain [95], [^11^C]LSN3172176 was advanced to human studies for further evaluation, which confirmed its promising properties as PET ligand to quantify mAChR M1 in the human brain [96].

The Lindsley group at Vanderbilt University has extensively optimized mAChR M4 positive allosteric modulators (PAMs) [97]. Eventually this led to the high affinity PAM VU0467485, which was deemed suitable as PET tracer candidate [98]. However, no specific binding could be observed in rat brain autoradiography in self-blocking experiments using [^11^C]VU0467485. Two closely related congeners, in which a single hydrogen was substituted with a fluorine, were also carbon-11 labeled and evaluated using autoradio-graphy. Interestingly, both showed a drastic increase in specific binding. [^11^C]M_4_R-1023, the congener with the highest specific binding as observed in autoradiography, was chosen for in vivo imaging of rats. Due to its low brain uptake [^11^C]M_4_R-1023 is no likely candidate for further preclinical evaluation; nevertheless, it may act as starting point for further chemical optimization [98].

[^11^C]MK-6884 is the most recently reported potential mAChR radiotracer studied in vivo and is under development by Merck & Co. It arose from optimization of binding affinity and balancing of physico-chemical properties from a lead series of potent M4 PAMs. In vivo imaging studies on monkeys showed rapid BBB penetration and activity distribution according to the known expression pattern of cerebral mAChR M4 with the highest uptake in the striatum. Intravenous injection of a different M4 PAM resulted in significant reduction of radiotracer binding, indicating specific binding. In vitro autora-diography on rat brain sections using a tritiated compound of a similar chemotype showed that the radiotracer only bound in presence of an agonist (carbachol) [99]. 

This supports that [^11^C]MK-6884 only binds to activated receptors; however, no in vivo study proofing or falsifying this hypothesis was performed. The first clinical study using [^11^C]MK-6884 was completed in 2019 and concluded that this tracer might be useful for imaging muscarinic receptors in Alzheimer’s disease [111].

One of the main lessons that can be learned from the last decades of mAChR radiotracer development is that physico-chemical properties have to be balanced carefully in order to receive a feasible compromise of signal-to-noise ratio, flow independency, non-specific binding, and BBB permeability.

### 2.3. PET Tracer Development for In Vivo Muscarinic Imaging of the PNS

With some exceptions the vast majority of in vivo mAChR imaging studies targets the CNS; although, mAChRs are also of high relevance in the PNS [122]. For example, *N*-methylated QNB ([^11^C]MQNB) was used to study mAChRs in human heart. Featuring a positively charged quaternary ammonium, [^11^C]MQNB is unable to penetrate the BBB and therefore can only bind to peripheral mAChRs [123]. Similarly, the positively charged and BBB impermeable compound [^11^C]VC-002 was recently used to image mAChRs in the lungs. In a first human study this tracer showed good repeatability and desired kinetic behavior, which paved the way for a further study aiming to estimate the mAChR occupancy in human lungs after inhalation of mAChRs antagonists [124]. So far, the high whole-body acquisition time due to the limited field of view of conventional PET scanners has made them inefficient to follow, with a dynamic acquisition protocol, the peripheral distribution of neuroimaging radiotracers. Considering the advent of full-body PET scanners [125], much more information on the peripheral mAChR radiotracer distribution may be available in the future. In view of the overexpression of mAChRs in a wide variety of cancer types [126], it is conceivable that mAChR PET radiotracers might also find application in cancer imaging. High expression of mAChR M3 recently was identified as a bio-marker in patients with non-small cell lung cancer [127] and mAChR M1 was shown to be involved in the migration and invasion of prostate cancer [128]. However, as of now, no in vivo studies imaging mAChRs as biomarkers in malignancies were reported.

## 3. Conclusions and Perspective

The last decades have given rise to several potential small molecule PET tracers aiming to image mAChRs in the CNS and PNS. Many of these compounds failed already in preclinical development. Flow dependent tracer accumulation hampering accurate and reliable mAChR imaging appears as the most common challenge for this molecular target, but also limited specific binding, metabolic instability, and inability to cross the BBB represent considerable hurdles in mAChR PET tracer development. The vast majority of clinical mAChR PET scans have been recorded using non-subtype selective ligands, which gives only an undifferentiated picture of the mAChR biology. Due to the similarities in the orthosteric binding pockets of mAChR M1-M5, development of subtype selective drugs remains a challenging task, but subtype selectivity has become a pivotal characteristic of newly developed mAChR ligands to reveal the distinct biological roles of the mAChR subtypes. Until recently allosteric mAChR ligands with sufficient affinity (K_i_ in the low nanomolar range) were not available [6]. Recent advances in crystallography of the mAChR enabled rationalized development of allosteric mAChR ligands, which eventually led to allosteric ligands with sufficient affinity for PET tracer development (M_4_R-1023 and MK-6884). With respect to subtype-selectivity allosteric ligands appear to be a promising game-changer for mAChR PET tracer development. The vast majority of reported mAChR tracers focus on brain imaging. Regarding the few examples of peripheral mAChR imaging, only heart and lungs have investigated. To the best of our knowledge, so far, no subtype-selective tracers have been used to study peripheral mAChRs. Considering the advent of whole-body PET, deeper insights into mAChRs in the periphery might be gathered as secondary findings of CNS scans. The majority of described potential mAChR PET tracers are radiolabeled with carbon-11. Although carbon-11 certainly is a highly desirable PET nuclide with respect to authentic labeling and low radiation burden, PET nuclides with a longer half-life have the potential to compensate for flow-dependent tracer distribution, which is a frequently observed challenge in mAChR tracer development. In addition to a majority of non-subtypeselective mAChR tracers, tracers with in vivo selectivity for the subtypes M1 ([^11^C]GSK1034702, [^11^C]LSN3172176), M2 ([^18^F]FP-TZTP) and M4 ([^11^C]MK-6884) have been reported and used for human brain imaging using PET. However, to the best of our knowledge no tracer targeting M3 or M5 is even at an early stage of development. Still, such radioligands are highly appreciated to provide a more complete understanding of the role of mAChRs in human physiology and disease by molecular imaging in the future.

## Figures and Tables

**Table 1:** Design and test criteria for the discovery and development of small molecule radiotracers [36].

Design Criteria	Test Criteria
Choosing an appropriate target High affinity and selectivity for the target Ease of radiosynthesis Maximizing target accessibility while minimizing non-displaceable binding	Good signal-to-noise ratio in vivo Good in vivo pharmacokinetics In vivo distribution and pharmacology consistent with literature reports Low levels of radiolabeled metabolites in the region of interest High sensitivity toward the target

**Table 2:** Binding parameters of well-known mAChR ligands given in nM. Data of scopolamine is given as K_i_ values from a competitive radioligand binding assay using [^3^H]NMS. Values of BQCA are given as inflection point of ACh efficacy potentiation as measured by calcium mobilization.

Compound	Binding Site	M1	M2	M3	M4	M5	lit.
Scopolamine	orthosteric	1.1	2.0	0.44	0.8	2.07	[55]
BQCA	allosteric	845	>100,000	>100,000	>100,000	>100,000	[59]

**Table 3:** Binding affinities of molecules shown in Figure 2. Values are given in nM. Wherever available K_D_/K_i_ values on membranes of transfected cells are reported.

Trivial Name	Systematic Name	M1	M2	M3	M4	M5	Method
(*R*,*S*)-IQNB	(*R*)-quinuclidin-3-yl (S)-2-hydroxy-2-(4-iodophenyl)-2-phenylacetate	0.49	-	1.27	-	-	K_D_ on transfected A9 L cell membranes using (R,S)-[^125^I]IQNB [112]
QNB	quinuclidin-3-yl 2-hydroxy-2,2-diphenylacetate	0.044	0.030	0.080	0.037	0.065	K_D_ on transfected CHO-K1 cell membranes using [^3^H]QNB [113]
scopolamine	(1*R*,2*R*,4*S*,5*S*,7*s*)-9-Methyl-3-oxa-9-azatricyclo[3.3.1.0^2,4^]non-7-yl (2*S*)-3-hydroxy-2-phenylpropanoate	7.5	9.5	6.5	36.9	17.6	K_i_ on transfected CHO-K1 cell membranes using [^3^H]NMS [114]
benztropine	(1*R*,3*r*,5*S*)-3-(benzhydryloxy)-8-methyl-8-azabicyclo[3.2.1]octane	6.8	14.1	11.2	22.9	4.6	K_i_ on transfected Sf9 cell membranes using [^3^H]NMS [115]
TRB	(1*R*,3*r*,5*S*)-8-methyl-8-azabicyclo[3.2.1]octan-3-yl 2-hydroxy-2,2-diphenylacetate	0.7, subtypes were not discriminated					IC_50_ by [^3^H]QNB competitive binding on mouse brain homogenates [116]
NMPB	1-methylpiperidin-4-yl 2-hydroxy-2,2-diphenylacetate	0.41, subtypes were not discriminated					K_D_ on mouse cortex [117]
4-FDEX	(*S*)-1’-(4-fluorobenzyl)-3-phenyl-[3,4’-bipiperidine]-2,6-dione	98, subtypes were not discriminated					IC_50_ by [^3^H]NMS competitive binding on rat brain homogenates [118]
xanomeline	3-[4-(hexyloxy)-1,2,5-thiadiazol-3-yl]-1,2,5,6-tetrahydro-1-methylpyridine oxalate	7.9	8.1	7.8	11.2	9.3	K_i_ on transfected CHO-K1 cell membranes using [^3^H]NMS [119]
FP-TZTP	3-(3-fluoropropylsulfanyl)-4-(1-methyl-3,6-dihydro-2*H*-pyridin-5-yl)-1,2,5-thiadiazole	7.4	2.2	79.7	-	-	K_i_ on different tissues with different radioligands [106]
(+)-3-MPB	1-methylpiperidin-3-yl 2-hydroxy-2,2-diphenylacetate	1.7 no significant selectivity					K_i_ on rat neocortex with [^3^H]QNB. No significant subtype selectivity was observed on transfected CHO-K1 cell membranes using a direct radioligand binding assay [120].
GSK1034702	4-fluoro-6-methyl-1-(1-(tetrahydro-2*H*-pyran-4-yl)piperidin-4-yl)-1,3-dihydro-2*H*-benzo[*d*]imidazol-2-one	7.9	>790	>790	>790	>790	EC_50_ of FLIPR assay of stably transfected CHO cells [88]
AF150(S)	2-methyl-8-methyl-1-thia-3,8-diazaspiro[4.5]dec-2-ene	390	22,000	-	-	-	K_i_ on rat cerebral cortex using [^3^H]pirenzepine (M1) or rat cerebellum using [^3^H]QNB (M2) [121].
-	(*S*,*R*)-1-methylpiperidin-3-yl 2-cyclopentyl-2-hydroxy-2-phenylacetate	3.5	-	-	-	-	K_i_ of “high affinity human mAChR M1 assay”. A degree of M1 selectivity was evident from partial blocking of the radioligand with pirenzepine in autoradiography using human brain slices [94].
LSN3172176	ethyl 4-(6-methyl-2-oxoindolin-1-yl)-[1,4’-bipiperidine]-1’-carboxylate	8.9	63.8	3031	41.4	55.6	K_i_ on transfected CHO-K1 cell membranes using [^3^H]NMS [89].
M_4_R-1023	5-amino-*N*-(2,3-difluoro-4-methoxybenzyl)-3,4-dimethylthieno[2,3-*c*]pyridazine-6-carboxamide	43.4	>10^4^	>10^4^	>10^4^	>10^4^	EC_50_ of calcium release assay on stably transfected CHO cells [97].
MK-6884	6-(2-methyl-3-oxoisoindolin-5-yl)-5-(1-((1-methylcyclopentyl)methyl)-1*H*-pyrazol-4-yl)picolinonitrile	-	-	-	0.19	-	K_i_ on transfected CHO-K1 cell membranes using a tritiated compound of similar chemotype [99]

## Data Availability

Data sharing not applicable.
